# Glacial expansion of cold-tolerant species in low latitudes: megafossil evidence and species distribution modelling

**DOI:** 10.1093/nsr/nwad038

**Published:** 2023-02-15

**Authors:** Luliang Huang, Shufeng Li, Weiye Huang, Helanlin Xiang, Jianhua Jin, Alexei A Oskolski

**Affiliations:** State Key Laboratory of Biocontrol and Guangdong Provincial Key Laboratory of Plant Resources, School of Life Sciences/School of Ecology, Sun Yat-sen University, China; State Key Laboratory of Palaeobiology and Stratigraphy, Nanjing Institute of Geology and Palaeontology, Chinese Academy of Sciences, China; CAS Key Laboratory of Tropical Forest Ecology, Xishuangbanna Tropical Botanical Garden, Chinese Academy of Sciences, China; State Key Laboratory of Biocontrol and Guangdong Provincial Key Laboratory of Plant Resources, School of Life Sciences/School of Ecology, Sun Yat-sen University, China; State Key Laboratory of Biocontrol and Guangdong Provincial Key Laboratory of Plant Resources, School of Life Sciences/School of Ecology, Sun Yat-sen University, China; State Key Laboratory of Biocontrol and Guangdong Provincial Key Laboratory of Plant Resources, School of Life Sciences/School of Ecology, Sun Yat-sen University, China; Department of Botany and Plant Biotechnology, University of Johannesburg, South Africa; Botanical Museum, Komarov Botanical Institute of the Russian Academy of Sciences, Russia

## Abstract

Fossil wood of Chinese white pine (Pinus armandii Franch.) from the Late Pleistocene deposits of Maoming Basin of South China provides the first megafossil evidence for glacial expansion of the range of a cold-tolerant species in low latitudes.

The Pleistocene was a time of repeated glaciations that severely affected the land biota over large areas of Eurasia and North America. Drastic climatic fluctuations during the Pleistocene drove large-scale extinctions, migrations and diversification in plant and animal populations. These dramatic processes had a strong impact on the distribution of modern flora and fauna.

Pleistocene climate oscillations have been expressed differently in various regions depending on the distance from the equator, ocean position and currents, mountain ranges, etc. [1]. Although the most prominent environmental and biotic changes occurred in the glaciated zones of high- and mid-latitudes, the low-latitude regions were also influenced by these climatic fluctuations [2]. The multiple effects of Pleistocene glaciations on the climate and plant diversity in tropical and subtropical regions are still poorly known due to insufficient data and controversial results obtained from different geological and paleontological proxies, as well as climate models. Further exploration of the effects is important not only for better reconstruction of the past, but also for climate forecasting and conservation of plant diversity in the context of present global climate changes [3].

The distributions of many species contracted during the glaciation with persistence confined to refugia with favourable habitats followed by range expansion during interglacials. This scenario, known as the interglacial expansion hypothesis [4] or expansion-contraction model [5], was not the only possible response to Pleistocene climatic fluctuations. Instead, glaciations provide favourable conditions under which some cold-tolerant species were able to expand during the cold periods, and then contract their ranges to the interglacial refugia [6]. Plant species with Arctic-alpine distributions in Europe and North America, such as *Dryas octopetala* L., *Dryas integrifolia* Vahl and *Salix herbacea* L. [7,8], show the obvious confirmation of this glacial expansion model [9] supported by rich megafossils and pollen records, as well as by molecular evidence. This scenario has also been suggested by molecular phylogeographic studies of several plant species distributed in low latitudes of eastern Asia, such as *Pinus armandii* Franch. [10,11] and *Rosa sericea* Lindl. [9]. No fossil evidence of glacial range expansion for plant species with low-latitude distributions has been reported to date.

The Chinese white pine (*Pinus armandii*) is a coniferous tree distributed in the Qingling Mountains, Daba Mountains and terrains in the Yunnan-Guizhou Plateau of central and southwest China, with one variety (*Pinus armandii* var. *mastersiana* (Hayata) Hayata) extending to Taiwan. This large-seeded and animal-dispersed pine is a typical cold-tolerant mountain species occurring across an elevation of 1000–3300 m [10] (Fig. 1A). Molecular phylogeographic analysis of *Pinus armandii* and related species of white pines suggests the important role of isolation and fragmentation during Pleistocene climatic oscillations for genetic diversification of this group [10].

**Figure 1.**
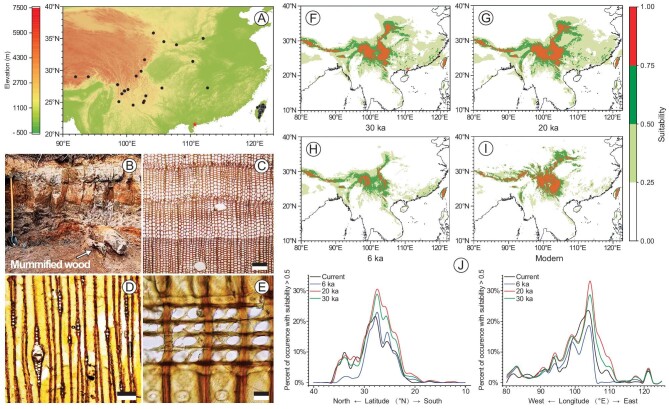
(A) The extant distribution of *Pinus armandii* (including *Pinus armandii* var. *mastersiana*) (black dots) and the fossil locality (red star). (B) Stratigraphic section of the Upper Pleistocene in the Maoming Basin. (C) Transverse section showing distinct growth rings with gradual to somewhat abrupt transitions from earlywood to latewood; axial resin canals in latewood; scale bar = 200 μm. (D) Tangential section; rays are mostly uniseriate and occasionally fusiform; scale bar = 100 μm. (E) Radial section; cross-field pits are mainly ‘window-like’ (fenestriform), ray parenchyma with smooth horizontal and end walls; scale bar = 20 μm. (F) Projected climatically suitable habitats for *Pinus armandii* at 30 ka; the red star indicates the fossil locality. (G) Projected climatically suitable habitats for *Pinus armandii* at 20 ka. (H) Projected climatically suitable habitats for *Pinus armandii* at 6 ka. (I) Projected suitable habitats under current climate. (J) The range shift of suitable habitats with suitability > 0.5 of *Pinus armandii* in latitude and longitude at different times.

In this study, we present anatomical investigations of the mummified wood of *Pinus* L. from the Upper Pleistocene (i.e. 33–30 ka cal. BP) of  Maoming Basin, Guangdong Province, South China (Fig. 1A–E; Fig. S1). Detailed comparison with available data on the wood structure of this genus strongly suggests its greatest affinity to the extant *Pinus armandii*. This determination has been confirmed by the anatomical examination of modern and extinct wood of this genus (Fig. S2; Table S1). This is direct evidence for the wider-than-present range of this species during the last glaciation prior to the Last Glacial Maximum (LGM). Paleoclimatic data from Coexistence Approach (CA) data, Oscillayers and the WorldClim data set (Tables S2 and S3), combined with the fossil record and the results from the Biomod2 ensemble species distribution models (SDMs), strongly indicated a cool and dry climate in low latitudes during the last glaciation and a glacial expansion scenario for the recent biogeographical history of *Pinus armandii*. The mummified wood is the first megafossil evidence for this scenario explaining species distributions in low latitudes, and sheds light on how cold-adapted organisms in low latitudes responded to Quaternary climatic fluctuations.

The CA data based on the co-occurrence of four species from Maoming (*Pinus armandii, Pinus hwangshanensis* W.Y. Hsia, *Keteleeria davidiana* (Bertr.) Beissn. and *Liquidambar formosana* Hance) (Table S2), coupled with the results of paleoclimatic reconstructions based on Oscillayers data sets (Table S3), strongly suggest that the Late Pleistocene (30 ka) climate of the Maoming Basin was characterized by lower temperatures with more prominent temperature seasonality (BIO4) than experienced today in this region. The Oscillayers and WorldClim estimations for this locality also suggest that the climate in the Late Pleistocene was drier, but with more winter precipitation than in the Holocene (Table S3). The fossil data do not provide much additional precipitation data, but they detect higher precipitation during the dry season and, therefore, lower precipitation seasonality than other proxies suggest. All incongruousness between the CA ranges and the estimations based on climate modelling are associated with the narrower ranges of respective climatic parameters in *Pinus hwangshanensis* than in the other three species included in the CA analysis. The climate condition of Maoming during the Late Pleistocene is in agreement with that of other areas of South China based on previous palynological studies, which show a significantly colder and dryer climate than present [12].

The occurrence of the *Pinus armandii* wood in the Late Pleistocene of the Maoming Basin shows that the range of this species prior to the LGM extended to South China. Biomod2 ensemble SDMs show that in the last glaciation (30 ka and 20 ka) climatically suitable habitats for *Pinus armandii* expanded southward (even reaching South China) and eastward compared to the Mid-Holocene (6 ka) and the present (Fig. 1F–J). Thus, our fossil report of this species, combined with the results of Biomod2 ensemble SDMs, demonstrates its occurrence in this region in the Late Pleistocene as well as a fast contraction of its distribution area during the Holocene. Apparently, the cooler and more continental climate of the Maoming Basin in the Late Pleistocene (Table S3) was favourable for the lowland distribution of this relatively cold-adapted montane species.

Results of previous studies suggest that temperature factors have the greatest influence on the extant distribution of *Pinus armandii* [13,14]. Our Biomod2 ensemble SDM results (Figs S3–S5) also show that the five most important bioclimatic variables influencing the distribution of *Pinus armandii* are minimum temperature of coldest month (BIO6), maximum temperature of warmest month (BIO5), temperature seasonality (BIO4), mean diurnal range (BIO2) and annual mean temperature (BIO1) (Fig. S4), while precipitation was the least important factor (Figs S4 and S5). These data are perfectly consistent with the results of experimental assessments of the influence of temperature on seed germination and the early development of seedlings in different populations of *Pinus armandii*, which suggest that the extreme (i.e. minimum and maximum) annual temperatures are the critical parameters affecting reproduction of this species [15].

Notably, the values of BIO1, BIO4 and BIO5 estimated by different paleoclimatic proxies (Table S3) for the period from the Late Pleistocene to the present do not exceed the limits of the ranges of respective bioclimatic parameters within the modern distribution area of *Pinus armandii* (Table S2). The mean diurnal range (BIO2) and the minimum temperature of the coldest month (BIO6) estimated for past climates (including that for the Mid-Holocene) are also within the ranges of this species, but their modern values for the fossil locality in the Maoming Basin (7.1°C for BIO2 and 11.8°C for BIO6) are close to the lower limit of BIO2 (6.8°C) and the upper limit of BIO6 (13.2°C) for *Pinus armandii*, respectively (Fig. S6). These data suggest that the decrease of daily range of temperature (BIO2) and the warming of winters (BIO6) during the Holocene might be two of the most influential climatic factors that drove the retreat of this cold-tolerant species from the Maoming Basin.

Our data are consistent, therefore, with a scenario which includes the glacial expansion of the range of *Pinus armandii* during the Late Pleistocene and its subsequent contraction to interglacial refugia [6] in the mountains of central and southwest China during the Holocene. Although this scenario has been suggested by MaxEnt SDM modelling for a group of East Asian white pines (including *Pinus armandii*) [11], no fossil records or other SDMs confirming such range shifts associated with climate change during the Quaternary have been reported, from low latitudes. Previous palynological studies from this area mostly highlighted paleovegetation shifts along with climate change during the Quaternary [12], whereas the glacial expansion and interglacial contraction of the ranges of plant species had not been reported yet. The wood of *Pinus armandii* from the Late Pleistocene of the Maoming Basin is the first megafossil, combined with the Biomod2 ensemble SDM, confirming the glacial south-eastern range expansion of a cold-tolerant high-elevation species in a subtropical region prior to the LGM and interglacial contraction during the Holocene. The scenario of glacial expansion suggested in this study for *Pinus armandii* might be common for other montane cold-tolerant plants and other organisms distributed in subtropical and tropical regions.

## Supplementary Material

nwad038_Supplemental_File
