# Supplementary material for: Glacial expansion of cold-tolerant species in low latitudes: megafossil evidence and species distribution modelling
Source: Natl Sci Rev. 2023 Feb 15;10(4):nwad038. doi: 10.1093/nsr/nwad038 (PMC10029839; doi:10.1093/nsr/nwad038)
Supplement: nwad038_Supplemental_File [file nwad038_supplemental_file.docx]

**Supplementary Files:**

**Material and Methods**

**Systematic paleontology**

**Discussion**

**Figures: 6**

**Tables: 3**

**References: 57**

**1 Materials and Methods**

- 1. **Geological setting**

The fossil woods studied here were collected from a recently discovered deposit within the Maoming Basin, near Zhenjiang Town of Maoming City, Guangdong Province, South China (21°52′47.5′′ N; 110°40′06.3′′E, Fig. 1A; in text).

The Maoming Basin is located in southwestern of Maoming, Guangdong Province, and hosts one of the largest, mainly Cenozoic, oil shale deposits in China (Nan and Zhou, 1996). Plant fossils from Maoming Basin are mainly known from Paleogene deposits (Herman et al., 2017; Jin et al., 2017), and rarely discovered from Quaternary of Maoming. Recently, a new fossiliferous deposit, lying unconformably on the upper Eocene Huangniuling Formation of Maoming Basin, has been discovered (Huang et al., 2021a) yielding many mummified fossil seeds, fruits, and woods. The deposit is mainly composed of yellow, gray, and black mudstones, grayish yellow and grayish-white fine sandstones, with conglomerate and its geological age is the Late Pleistocene (29–27 ka BP, 33–30 ka cal. BP). i.e., MIS (Marine oxygen isotope stage) 3a according to Accelerator Mass Spectrometry (AMS) ^14^C dating for the plant fossils from this stratum (Huang et al., 2021a).

The fossil woods in this study were well-preserved by mummification (i.e. minimally altered from their original state), maintaining the cells and tissue systems that were there when the plant was alive (Taylor et al., 2015). A unique combination of lithological features, deposition rate of surrounding rocks, moisture and temperature, is required for such an unusual way of wood fossilization (Taylor et al., 2009).

**1.2 Climatic Data Acquisition**

**1.2.1 Data for Coexistence Approach**

We used the Coexistence Approach (CA) (Utescher et al., 2014) to quantify the ranges of climatic factors for the species represented by fossil plants known from the Upper Pleistocene of the Maoming Basin. We assessed the average, minimum and maximum values of 19 bioclimatic parameters for the distribution ranges of *Pinus armandii* Franch., *Pinus hwangshanensis* W.Y. Hsia, *Liquidambar formosana* Hance, and *Keteleeria davidiana* (Bertr.) Beissn.; all species reported to date are from this site (Huang et al., 2021a; Huang et al., 2021b; Bazhenova et al., 2022, present study). Because the fossil wood of *Keteleeria* from the Upper Pleistocene of the Maoming Basin has not been attributed to any modern species (Huang et al., 2021a), we analyzed the data for *Keteleeria davidiana* as its range extends further northwards than other members of this genus. The values of bioclimatic variables were revealed on the coordinates of 914 species occurrences retrieved from the Global Biodiversity Information Facility (GBIF) web portal (<http://www.gbif.org/>). The number of occurrences of these four species are 122 in *Keteleeria davidiana*, 706 in *Liquidambar formosana*, 65 in *Pinus armandii*, and 21 in *Pinus hwangshanensis.* This data set was used to extract 19 bioclimatic variables representing the variation of temperature and precipitation from the WorldClim climate layers at a resolution of 2.5 arc-min (Hijmans et al., 2005) for each occurrence using the Raster package (Hijmans and van Etten, 2012) in R.

**1.2.2 Data from other sources**

The Oscillayers dataset (Gamisch, 2019) was downloaded from the Dryad Digital Repository, from which high spatial (2.5 arc-minutes) and temporal resolution (10 Kyr time periods) paleoclimatic data from 2.58 Ma to 20 Kyr can be obtained. Paleoclimate data for the fossil locality (paleo-coordinates were reconstructed using the Chronosphere package) were retrieved by the “bilinear” method of Raster package in R as previously described (Kocsis and Raja, 2021; Hijmans et al., 2022).

Bioclimatic variables of modern (1960−1990) and Mid-Holocene (6 ka) were downloaded from WorldClim version 1.4 (Hijmans et al., 2005) at a resolution of 2.5 arc-min.

**1.3 Biomod2 Ensemble Species Distribution Modeling**

The nineteen bioclimatic variables for both modern and paleo-climate obtained from WorldClim (Fick and Hijmans, 2017) (6 ka, modern) and Oscillayers (Gamisch, 2019) (30 ka, 20 ka) were used to compare with the CA bioclimatic variables at the fossil locality and model the potential ancient species distribution of *Pinus armandii* in southeastern Asia (longitude: 80°E−123°E, latitude: 10°N−40°N) by means of the Biomod2 R package (Thuiller et al., 2021). From Oscillayers dataset, we chose the bioclimatic variables since 30 ka, the time closest to the age of fossil locality. Biomod2 combines various statistic-based and machine learning-based SDM algorithms to optimize the habitat suitability estimation (Hastie et al., 1994; Araújo and Guisan, 2006; Leathwick et al., 2006). Species occurrence data were collected from the Global Biodiversity Information Facility (http://www.gbif.org/) and Conifers of the world (http://herbaria.plants.ox.ac.uk/bol/conifers/Explore).

**1.3.1 Climatic variable processing**

Bioclimatic variables with greater degree of correlation (high multicollinearity) among themselves could affect the performance of model (Dormann et al., 2013). Thus, we removed variables with Pearson correlation coefficient greater than 0.96 and taking into consideration the empirical knowledge about the importance of climate variables. After this selection procedure, 15 variables were retained for modelling the distribution of *Pinus armandii*: Annual mean temperature (BIO1, ℃), Mean diurnal range (BIO2, ℃), Isotermality (BIO3), Temperature seasonality (BIO4), Max. temperature of warmest month (BIO5, ℃), Minimum temperature of coldest month (BIO6, ℃), Temperature annual range (BIO7, ℃), Mean temperature of wettest quarter (BIO8, ℃), Mean temperature of driest quarter (BIO9, ℃), Annual precipitation (BIO12, mm), Precipitation of wettest month (BIO13, mm), Precipitation of driest month (BIO14, mm), Precipitation seasonality (BIO15), Precipitation of warmest quarter (BIO18, mm), Precipitation of coldest quarter (BIO19, mm). The final climate variables were then selected according to the variable importance (a random forest-based variable importance algorithm as described in Breiman (2001)) results from a preliminary Biomod2 run. The four climate variables with relative low importance values were then removed from the above 15 variables, i.e., BIO14, BIO15, BIO13, BIO8.

**1.3.2 Model establishment**

The ensemble model was run initially with ten algorithms, including Artificial Neural Networks (ANN), Surface Range Envelope (SRE), Flexible Discriminant Analysis (FDA), General Linear Models (GLM), General Additive Models (GAM), General Boosted Models (GBM), Classification Tree Analysis (CTA), Multiple Adaptive Regression Splines (MARS), Random Forests (RF), and Maximum Entropy (MaxEnt. Phillips.2) model. Four models were finally selected for the run according to ROC (Receiver Operating Characteristics) (>0.85) and TSS (True Skill Statistics) thresholds (>0.80), i.e., “GLM”, “CTA”, “MARS”, “MAXENT.Phillips.2”.

A random selection of 80% of the modern presence-pseudoabsence data of *Pinus armandii* (retrieved from GBIF as described above) was used to train each SDMs 10 times (for a total of 100 runs for the 10 algorithms), and the remaining 20% was used for cross-validating, with ROC, TSS measured for evaluation (Allouche et al., 2006; Breiner et al., 2018).

The ensemble SDM was used for projecting the modern and past suitable habitat of *Pinus armandii*, including at 30 ka (the present fossil record), 20 ka (Last Glacial Maximum, LGM), 6 ka (Mid-Holocene) and 0 ka (modern).

To quantify the range shift of suitable habitats of *Pinus armandii* in terms of longitude and latitude, raster cells with suitability greater than 0.5 at each latitude (or longitude) were counted and scaled by dividing by the total number of raster cells at that latitude (or longitude). The range shifts in different periods were then plotted and smoothed using a loess (locally weighted regression) algorithm in OriginPro for visualization.

**2 Systematic paleontology**

Order **Coniferales** Gorozh.

Family **Pinaceae** Lindl.

Genus ***Pinus*** L.

Subgenus ***Strobus*** (D. Don) Lemmon

Species ***Pinus armandii* Franch.** (Fig. S1)

**Material:** MMHW020, 027, 034, 035; well-preserved mummified fossil wood

**Locality and age:** Recovered from the Upper Pleistocene of the Maoming Basin, Guangdong Province, South China; Late Pleistocene (29–27 ka BP, 33–30 ka cal. BP).

**Remarks:** Apart from the fossil samples, we examined the structure of modern woods of *Pinus armandii* Franch. and *Pinus koraiensis* Sieb. et Zucc. to clarify some anatomical traits of diagnostic importance. The wood samples of these species were obtained from Wood Collection of Chinese Academy of Forestry (catalogue No. 11231, 18083). Terminology used for the wood anatomical descriptions in this paper follow the recommendations of the International Association of Wood Anatomists (IAWA) list of Microscopic Features for Softwood Identification (IAWA Committee, 2004).

**Description:** Growth rings distinct, 0.51–1.80 mm wide (mean 1.18 mm, n=30), transition from earlywood to latewood is slightly gradual to abrupt (Fig. S1A). Earlywood tracheids square, polygonal, oval in outline, 12.64–64.8 μm (mean 35.3 ± 0.85 μm) in tangential diameter and 14.1–78.1 μm (mean 39.7 ± 0.98 μm) in radial diameter; thin-walled, 1.4–4.3 μm (mean 2.6 ± 0.05 μm) in thickness. Latewood tracheids polygonal to oval and tangentially elongated in outline, 6.2–56.9 μm (mean 26.2 ± 0.88 μm) in tangential diameter and 2.4–45.2 μm (mean 20.0 ± 0.71 μm) in radial diameter; relatively thick-walled, 2.1–5.1 μm (mean 3.5 ± 0.06 μm) in thickness.

The bordered pits on radial tracheid walls are mainly uniseriate, occasionally biseriate (opposite) (Fig. S1B). The pits are circular to oblong oval in outline, with 12.3–28.7 μm (mean 20.1 ± 0.26 μm) in tangential diameter. Crassulae are present. Helical thickenings are absent.

Axial parenchyma is absent.

Rays are uniseriate and fusiform (Fig. S1C), mainly composed of ray parenchyma; ray tracheids are few. Uniseriate rays are 1–15 cells (mean 5 cells) in height, 6.2–12.5 μm (mean 10.9 ± 0.23 μm) in width. Fusiform rays are 31.7–66.0 μm (mean 48.5 ± 1.48 μm) in width, biseriate with a single resin canal occurring in the central portion (Fig. S1D). Horizontal walls of ray parenchyma are relatively thick, few pitted; the end walls of ray parenchyma are smooth (Fig. S1E, F). Ray tracheids are occasionally in one tier at the margins of rays, with smooth horizontal walls and pitted end walls (Fig. S1E). Cross-field pits are mainly “window-like” (fenestriform), sometimes pinoid; 1–2 pits (mostly 2) per cross field (Fig. S1F).

Both axial and radial resin canals are present (Fig. S1A, D). Axial resin canals are 64.8–304.8 μm (mean 142.9 ± 5.1 μm) in diameter. Radial resin canals are 31.7–66.0 μm (mean 48.5 ± 1.48 μm) in diameter.

**3 Discussion**

**3.1 Comparison with modern woods**

The fossil wood under study is characterized by the presence of both axial and radial resin canals with thin-walled epithelial cells, and the smooth horizontal walls of ray tracheids which strongly support its assignment into subgenus *Strobus* (D. Don) Lemmon (subgenus *Haploxylon* (Koehne) Rehder, the white or soft pines) in the genus *Pinus* (Phillips, 1941; Budkevich, 1961; Jiang et al., 2010). An InsideWood (2004-onwards) search for the combination of smooth ray tracheid cell walls (81p), window-like cross-field pitting (90p), presence of axial (109p) and radial canals (110p) returns the following modern species: *Pinus albicaulis* Engelm., *Pinus armandii*, *Pinus cembra* L., *Pinus fenzeliana* Hand.-Mazz., *Pinus flexilis* James, *Pinus koraiensis* Siebold et Zucc., *Pinus lambertiana* Douglas, *Pinus monticola* Douglas ex D.Don, *Pinus parviflora* Siebold & Zucc., *Pinus peuce* Griseb., *Pinus pumila* (Pall.) Regel, *Pinus sibirica* Du Tour and *Pinus strobus* L. All these species belong to the subsection *Strobus* of the section *Quinquefoliae*, subgenus *Strobus.* Unlike the sample under study, *Pinus cembra*, *Pinus flexilis* and *Pinus parviflora* share the cross-field pits occasionally arranged in two horizontal rows (Greguss, 1955; Budkevich, 1961; Schoch et al., 2004; InsideWood, 2004-onwards); our observations (Fig. S2A) and Lee and Eom’s (1988) data suggest that this feature occurs also in *Pinus koraiensis*. Then, *Pinus lambertiana* and *Pinus parviflora* are distinctive in the occurrence of numerous (up to 4) pits on cross-fields (Budkevich, 1961; Kellogg et al., 1982; InsideWood, 2004-onwards). Unlike the fossil wood, *Pinus albicaulis*, *Pinus fenzeliana* and *Pinus monticola* share mostly nodular end walls of ray parenchyma cells. Apart from that, the first two species as well as *Pinus flexilis*, *Pinus peuce* and *Pinus strobus* are distinctive in having broad (usually 4-seriate) fusiform rays (Budkevich, 1961; InsideWood, 2004-onwards). Finally, *Pinus pumila* and *Pinus sibirica* differ from the fossil wood by having exclusively uniseriate pitting on radial tracheid walls (Greguss, 1955; Budkevich, 1961; FFPRI Wood Database, 2014-onwards). Apart from that, the former species has distinctly shorter rays (up to 10 cells in height) than the sample under study (Benkova and Schweingruber, 2004).

As for other species of the subsection *Strobus* occurring in China, *Pinus kwantungensis* Chun and Tsiang differs from the sample under study by having mostly nodular end walls of the ray parenchyma cells (Jiang et al., 2010). Unlike those, *Pinus wallichiana* A. B. Jacks. has smooth tangential ray cell walls, but this species is different from the sample from the Maoming Basing in having cross-fields pits arranged in two horizontal rows (Esteban et al., 2004). *Pinus morrisonicola* Hayata is distinctive in having numerous (up to 4) pits on cross-fields and in the occurrence of piceoid pits among them (Kanehira, 1921; InsideWood, 2004-onwards). Finally, the available data on the wood structure of *Pinus armandii* are discrepant: the end walls of its ray cells are reported either as pitted (Greguss, 1955; InsideWood, 2004-onwards), or as pitted and smooth (Esteban et al., 2004), or as smooth (Budkevich, 1961; Jiang et al., 2010). Our observations on the extant wood sections of this species (Fig. S2B) confirmed the end walls of its ray parenchyma cells as being smooth whereas few pits occasionally occur on their horizontal walls. These traits suggest, therefore, that the wood from the Upper Pleistocene of the Maoming Basin shows the greatest similarity to *Pinus armandii*.

**3.2 Comparison with other fossil woods**

The Pleistocene *Pinus* wood from Maoming is characterized by the presence of both axial and radial resin canals with thin-walled epithelial cells, weakly dentate horizontal walls of ray parenchyma and the “window-like” cross-field pits. Within the *Pinus* or *Pinuxylon* fossil woods having smooth or weakly dentate horizontal walls of ray parenchyma (Table S1), the “window-like” cross-field pits have only been reported in the fossil woods of *Pinus albicauloides* S.K. Choi et K. Kim from the Miocene of Yamagata Prefecture (Choi et al., 2010); *Pinuxylon zobelianum* (Goeppert) Kräusel from the Miocene of Limburg, Netherlands (Van der Burgh, 1964); *Pinuxylon cembraeforme* Rössler from the Pliocene of Steiermark, Austria (Rössler, 1937); *Pinus armandii* and *Pinus* cf. *armandii* from the Pliocene of Yunnan, China (Yi et al., 2002; 2005); *Pinus* cf. *tabulaeformis* Carrière and *Pinus cembra* L. from Early Pleistocene of Tegelen, Netherlands (Van der Burgh, 1974). However, *Pinus* cf. *tabulaeformis*, *Pinus cembra*, and *Pinuxylon cembraeforme* differ from the fossil wood from Maoming by their lack of pinoid cross-field pits. Moreover, the former two species possess narrower tracheids in the earlywood than the wood under study. Unlike the fossil sample from Maoming, *Pinus albicauloides* and *Pinuxylon zobelianum* lack the pinoid cross-field pits, and the latter species also has narrower tracheids in the earlywood.

Finally, *Pinus armandii* and *Pinus* cf. *armandii* differ from the sample under study in having numerous nodular thickenings on the horizontal walls of ray cells, as well as more numerous (up to 5) pits in cross-fields, occasionally arranged in two horizontal rows. This suite of wood traits suggests, however, that these fossil woods described by Yi et al. (2002, 2005) from the Pliocene of Yunnan, show fewer similarities to modern *Pinus armandii* than the Pleistocene wood from the Maoming Basin. Thus, the fossil wood under study can be convincingly attributed to *Pinus armandii* rather than to any other extant taxon.

**3.3 Paleoclimatic data from different proxies**

Table S2 shows the average, minimum and maximum values of the bioclimatic parameters for *Pinus armandii*, *Pinus* *hwangshanensis*, *Liquidambar formosana* and *Keteleeria davidiana* whose fossils were also found in the Upper Pleistocene of the Maoming Basin (Huang et al., 2021a, 2021b; Bazhenova et al., 2022). Although the fossil wood of *Keteleeria* from this locality has not been attributed to a particular species (Huang et al., 2021a), we analyzed bioclimatic data only for *Keteleeria davidiana*, the species showing significant overlap with both *Pinus armandii* and *Liquidambar formosana* in their modern distribution areas. Among two other species of *Keteleeria* Carr., the range of *Keteleeria fortunei* (Murr.) Carr. weakly overlaps that of *Pinus armandii*, whereas the *Keteleeria evelyniana* Mast. range has very limited overlap with the distribution area of *Liquidambar formosana* (Fang et al., 2009).

We estimated the climatic ranges for the Late Pleistocene (33–30 ka) in the Maoming Basin based on the overlapping modern distribution areas of these four co-occurring species (*Pinus armandii*, *Pinus hwangshanensis,* *Keteleeria davidiana* and *Liquidambar formosana*), i.e., using the Coexistence Approach (CA). We also calculated the values of bioclimatic variables using the values of all 19 bioclimatic variables for 30 ka and 20 ka reconstructed by the Oscillayers dataset at the closest locality to the fossil site (22.5°N, 112.5°E). Finally, we extracted the bioclimatic variables for the Last Glacial Maximum (21 ka), Mid-Holocene (6 ka) and modern climate at the fossil locality (21.86667°N; 110.66667°E) from the WorldClim dataset. All these data are collated in the Table S3.

The values of bioclimatic variables reconstructed by the Oscillayers for the age 30 ka are mostly within their CA ranges based on fossil data, with exceptions for BIO2, BIO3, BIO14, BIO15, and BIO17 (Table S3). The figures for the mean diurnal range of temperature (BIO2) and the isotermality (BIO3) estimated by Oscillayers are in a small excess of their CA ranges. At the same time, the precipitation of the driest month (BIO14) reconstructed by Oscillayers, as well as precipitation of the driest quarter (BIO17) are distinctly lower than their values estimated by the CA. Finally, Oscillayers show greater precipitation seasonality (BIO15) than the CA.

The comparison of different paleoclimatic proxies (Table S3) strongly suggest that in the Late Pleistocene (30 ka) in the Maoming Basin there was cooler and drier, with somewhat higher temperature seasonality, than the modern climate in this region. Temperature parameters (BIO1, BIO5, BIO6, BIO8, BIO9, BIO10 and BIO11) did not show significant changes during the Late Pleistocene (30–20 ka), but the temperature seasonality (BIO4) and annual range (BIO7) became weaker during this time. Warming occurred after the LGM, and temperature conditions of the Mid-Holocene (6 ka) were similar to modern ones. The values of some precipitation variables (BIO12, BIO13, BIO16, and BIO18) based on Oscillayers and WorldClim models show an increase in rainfall (especially in summer time) after the LGM, while the wide CA ranges of these parameters do not show any notable change. For other precipitation parameters (BIO14, BIO17, BIO19) and precipitation seasonality (BIO15), the paleoclimatic proxies do not suggest any unambiguous trends during the Late Pleistocene and Holocene.

**3.4 Model performance and suitable habitats of *Pinus armandii* in present and past**

Among the four algorithms (Fig. S3), the MaxEnt. Phillips. 2 model had the best performance (TSS=0.872, ROC=0.966), followed by GLM, MARS and CTA.

The results obtained for the percent contribution of climatic variables showed that the most important climatic variables affecting shifts in species distributions of *Pinus armandii* in the ensemble model were BIO6, BIO5, BIO4, BIO2, BIO1, BIO7, BIO3, BIO9, BIO19, BIO12, BIO13, BIO15, BIO18, BIO8, and BIO14 with importance scores of 0.52, 0.50, 0.46, 0.37, 0.27, 0.24, 0.18, 0.13, 0.11, 0.10, 0.08, 0.08, 0.08, 0.07, and 0.03, respectively (Fig. S4).

The response curves of 15 variables regarding *Pinus armandii* from MaxEnt. Phillips. 2, which represents the quantitative relationship between environmental variables and the habitat suitability, are shown in Fig. S5. Based on the response curves, the likely suitable ranges of BIO6 was from -15°C to 15°C if the threshold of 0.6 was chosen, while -5°C to 5°C was the most suitable values of BIO6 for *Pinus armandii*. When BIO5 is larger than 30°C, habitat suitability will decease dramatically (threshold is 0.6). The habitat suitability shows negative relationships with BIO2 and BIO4. Precipitation shows less obvious relationship with habitat suitability except BIO15 and BIO19, the suitable values are likely less than 110 mm and 700 mm for these two climate variables respectively, by setting 0.6 as a threshold.

The Biomod2 ensemble model run for *Pinus armandii* (Fig. 1I, in text) showed that under today’s climate, highly climatically suitable habitats (suitability>0.5) were observed in extensively across central and southwest of China (including Shanxi, Henan, Shaanxi, Gansu, Sichuan, Hunan, Hubei, Guizhou, Yunnan, Tibet), and discontinuously extend to Taiwan, which matches closely the actual distribution of modern species. Additionally, a few areas such as Fujian, the places adjacent to Taiwan, also show climatically suitable for the growth of this species.

The projections of climatically suitable habitats for *Pinus armandii* at 30 ka and 20 ka (LGM) (Fig. 1F, G; in text), indicated that highly suitable habitats (suitability >0.5) were observed in addition to Taiwan, central and southwest of China, and extended to parts of southern China, such as Fujian, Zhejiang, Jiangxi, Guangxi and Guangdong. It is clear that climatically suitable habitats for this species during the last glaciation show a southward and eastward range expansion compared to the present (Fig. 1F, G, J; in text). The projected suitable habitats for our Maoming fossil locality, Guangdong, during this period show high suitability (0.3) compared to the present (0), indicating the relatively high survival possibility for *Pinus armandii* in these areas. When it comes to the Holocene, such as the Mid-Holocene (6 ka), suitable habitat areas (including Guangdong) show considerable contraction, except in Taiwan, with highly suitable habitats only observed in more limited areas in southwest of China compared to present and 30 ka, 20 ka (Fig. 1H, J; in text).

**References**

Allouche O, Asaf T and Ronen K. Assessing the accuracy of species distribution models: prevalence, kappa and the true skill statistic (TSS). *Journal of Applied Ecology* 2006; **43(6)**: 1223–32.

Araújo MB and Guisan A. Five (or so) challenges for species distribution modelling. *Journal of Biogeography* 2006; **33(10)**: 1677–88.

Benkova VR and Schweingruber FH. *Anatomy of Russian Woods. An atlas for the identification of trees, shrubs, dwarf shrubs and woody lianas from Russia*. Birmensdorf, Swiss Federal of institutes for Forest, Snow and Landscape Research. Berne, Stuttgart, Vienna, 2004.

Bazhenova NV, Wu XK, Kodrul TM *et al.* Mummified seed cones of *Pinus prehwangshanensis* sp. nov. (subgenus *Pinus*, Pinaceae) from the Upper Pleistocene of Guangdong, South China: taxonomical significance and implication for phytogeography and ecology. *Frontiers in Ecology and Evolution* 2022; **10**: 900687, doi: 10.3389/fevo.2022.900687.

Blackwell WH. Fossil Ponderosa-like pine wood from the Upper Cretaceous of north-east Mississippi. *Annals of Botany* 1984; **53(1)**: 133–6.

Blokhina NI. Petrified wood of pine *Pinuxylon chemrylensis* sp. nov. from the Paleogene of Kamchatka. *Paleontological Journal* 1995; **29(2A)**: 141–7.

Blokhina NI and Bondarenko OV. Fossil wood of *Pinus priamurensis* sp. nov. (Pinaceae) from the Miocene deposits of the Erkovetskii Brown Coal Field, Amur Region. *Paleontological Journal* 2016; **50(3)**: 311–8.

Budkevich EV. *Drevesina sosnovykh*. Izdatel’stvo Akademii Nauk SSSR, Moskva-Leningrad. 1961. (In Russian)

Breiman L. Random forests. *Machine Learning* 2001; **45 (1)**: 5–32.

Breiner F, Nobis MP, Bergamini A *et al.* Optimizing ensembles of small models for predicting the distribution of species with few occurrences. Edited by Nick Isaac. *Methods in Ecology and Evolution* 2018; **9**: 802–8.

Choi SK, Kim K, Jeong EK *et al.* Fossil woods from the Miocene in the Yamagata Prefecture, Japan. *IAWA Journal* 2010; **31(1)**: 95–117.

Dormann CF, Elith J, Bacher S *et al.* Collinearity: a review of methods to deal with it and a simulation study evaluating their performance. *Ecography* 2013; **36**: 27–46.

Esteban LG, de Palacios P, Casasús AG *et al.* Characterisation of the xylem of 352 conifers. *Investigación Agraria.* *Sistemas y Recursos Forestales* 2004; **13(3)**: 152–78.

Fang JY, Wang ZH and Tang ZY. *Atlas of Woody Plants in China*. *Distribution and Climate*. Vol. 1. Higher Education Press, Beijing; 2009.

Falcon-Lang H, Mages V and Collinson M. The oldest *Pinus* and its preservation by fire. *Geology* 2016; **44 (4)**: 303–6.

Forestry and Forest Products Research Institute (FFPRI) Wood Database. Matsunosato, Tsukuba, Ibaraki, Japan; 2014-onwards. http://f030091.ffpri.affrc.go.jp/index-E.

Fick SE and Hijmans RJ. WorldClim 2: new 1km spatial resolution climate surfaces for global land areas. *International Journal of Climatology* 2017; **37(12)**: 4302–15.

Gamisch A. Oscillayers: a dataset for the study of climatic oscillations over Plio-Pleistocene time-scales at high spatial-temporal resolution. *Global Ecology and Biogeography* 2019; **28**: 1552–60.

Greguss P. *Xylotomische Bestimmung der heute lebenden Gymnospermen*. Budapest: A Kademiai Kiado; 1955.

Hastie T, Tibshirani R and Buja A. Flexible discriminant analysis by optimal scoring. *Journal of the American Statistical Association* 1994; **89(428)**: 1255–70.

Herman AB, Spicer RA, Aleksandrova GN *et al*. Eocene–early Oligocene climate and vegetation change in southern China: evidence from the Maoming Basin. *Palaeogeography, Palaeoclimatology, Palaeoecology* 2017; **479**: 126–37.

Hijmans RJ, Cameron SE, Parra JL *et al.* Very high resolution interpolated climate surfaces for global land areas. *International Journal of Climatology* 2005; **25**: 1965–78.

Hijmans RJ and van Etten J. *raster: Geographic analysis and modeling with raster data*. R package version 2.0-12; 2012.

Hijmans RJ, van Etten J, Sumner M *et al.* *raster: Geographic data analysis and modeling*. R package version 3.6-3; 2022.

Huang LL, Jin JH and Oskolski AA. Mummified fossil of *Keteleeria* from the Late Pleistocene of Maoming Basin, South China, and its phytogeographical and paleoecological implications. *Journal of Systematics and Evolution* 2021a; **59(1)**: 198–215.

Huang LL, Jin JH, Quan C *et al.* New occurrences of Altingiaceae fossil woods from the Miocene and Upper Pleistocene of South China with phytogeographic implications. *Journal of Palaeogeography* 2021b; **10(4)**: 482–93.

IAWA Committee. IAWA List of microscopic features for softwood identification. *IAWA Journal* 2004; **25**: 1–70.

InsideWood. Published on the Internet; 2004–onwards. Available from http://insidewood.lib.ncsu.edu/search [accessed 5 August 2022].

Jiang XM, Cheng YM, Yin YF *et al.* *Atlas of Gymnosperms Woods of China*. Science Press, Beijing; 2010.

Jin JH, Herman AB, Spicer RA *et al.* Palaeoclimate background of the diverse Eocene floras of South China. *Science Bulletin* 2017; **62(2)**: 1501–03.

Jeong EK, Kim K, Suzuki M *et al.* Daijima-type coniferwood assemblage of the Hatamura Formation (Middle Miocene) in the Akita Prefecture, Japan. *Geosciences Journal* 2012; **16(2)**: 115–25.

Kanehira R. *Anatomical characters and identification of Formosan woods with critical remarks from the climatic point of view, with 300 micrographs*. Bureau of Productive Industries, Government of Formosa, Taihoku; 1921.

Kellogg RM, Rowe S, Koeppen RC *et al*. Identification of the wood of the soft pines of western North America. *IAWA Bulletin* n.s. 1982; **3 (2):** 95–101.

Kocsis ÁT and Raja NB. Chronosphere: *Earth System History Variable*s. Zenodo Version 0.4.1; 2021. https://doi.org/10.5281/zenodo.4746300.

Leathwick JR, Elith J and Hastie T. Comparative performance of generalized additive models and multivariate adaptive regression splines for statistical modelling of species distributions. *Ecological Modelling* 2006; **199(2)**: 188–96.

Lee PW and Eom YG. Anatomical comparison between compression wood and opposite wood in a branch of Korean pine (*Pinus koraiensis*). *IAWA Journal* 1988; **9(3)**: 275–84.

Miller CN. *Silicified cones and vegetative remains of Pinus from the Eocene of British Columbia*. Contributions from the Museum of Paleontology, the University of Michigan 1973; **24(10)**: 101–18.

Nan Y and Zhou GQ. *Stratigraphy (lithostratic) of Guangdong Province*. Wuhan: China University of Geosciences Press; 1996.

Nishida M and Nishida H. Pinoid woods with resin canals from the Upper Cretaceous of Hokkaido and Saghalien. *Journal of Plant Research* 1995; **108(2)**: 161–70.

Ogura Y. Notes on fossil woods from Japan and Manchoukuo. *Japanese Journal of Botany* 1944; **13**: 345–65.

Phillips EWJ. The identification of coniferous woods by their microscopic structure. *Botanical Journal of the Linnean Society* 1941; **52(343)**: 259–320.

Rössler W. Pliozäne Koniferenhölzer der Umgebung von Gleichenberg in Steiermark. *Mitteilungen des Naturwissenschaftlichen Vereines für Steiermark* 1937; **74**: 64–79.

Schoch W, Heler I, Schweingruber FH *et al.* Wood anatomy of Central European species; 2004. Online version: <http://www.woodanatomy.ch/>.

Stockey RA. *Pinus driftwoodensis* sp. n. from the Early Tertiary of British Columbia. *Botanical Gazette* 1983; **144(1)**: 148–56.

Tao JR, Yang JJ and Wang YF. Miocene wood fossils and paleoclimate in Inner Mongolia. *Acta Botanica Yunnanica* 1994; **16(2)**: 111–6. (In Chinese with English abstract)

Taylor EL, Taylor TN and Krings M. *Paleobotany: The Biology and Evolution of Fossil Plants. 2nd ed*. Academic Press, Holland, Amsterdam; 2009.

Taylor TN, Krings M and Taylor EL. *Fossil Fungi*. Academic Press, UK; 2015.

Thuiller W, Georges D, Gueguen M *et al.* Biomod2: Ensemble platform for species distribution modeling. R package version 3.5.1; 2021. [https://cran.r-project.org/pacKage=Biomod2](https://cran.r-project.org/package=biomod2).

Tidwell WD, Parker LR and Folkman VK. *Pinuxylon woolardii* sp. nov., a new petrified taxon of Pinaceae from the Miocene basalts of Eastern Oregon. *American Journal of Botany* 1986; **73(11)**: 1517–24.

Utescher T, Bruch AA, Erdei B *et al.* The Coexistence Approach-Theoretical background and practical considerations of using plant fossils for climate quantification. *Palaeogeography, Palaeoclimatology, Palaeoecology* 2014; **410**: 58–73.

Van der Burgh J. Hölzer der niederrheinischen Braunkohlenformation. 1. Hölzer der Braunkohlengrube “Anna” zu Haanrade (Niederländisch Lim-burg). *Acta Botanica Neerlandica* 1964; **13(2)**: 250–301.

Van der Burgh J. Hölzer der niederrheinischen Braunkohlenformation. 2. Hölzer der Braunkohlengruben “Maria Theresia” zu Herzogenrath, “ZukunftWest” zu Eschweiler und “Victor” (Zülpich-Mitte) zu Zülpich. Nebst einersystematisch-anatomischen Bearbeitung der Gattung *Pinus* L. *Review of Palaeobotany and Palynology* 1973; **15(2)**: 73–275.

Van der Burgh J. Wood-remains from the Lower Pleistocene of Tegelen (The Netherlands). *Scripta Geologica* 1974; **25**: 1–35.

Wang HB, Oskolski AA, Jacques F *et al.* Lignified woods of *Pinus* (Pinaceae) from the late Miocene of central Yunnan, China, and their biogeographic and paleoclimatic implications. *Palaeoworld* 2017; **26(3)**: 553–65.

Yang JJ, Qi GF and Xu RH. Studies on fossil woods excavated from the Dabie Mountains. *Scientia Silvae Sinicae* 1990; **28(4)**: 379–83. (In Chinese with English abstract)

Yi TM, Li CS, Jiang XM *et al.* Pliocene *Rhododendron* and *Pinus* fossil woods of Yunnan Province and palaeoclimate. *Journal of Palaeogeography* 2002; **4**: 90–8.

Yi TM, Li CS and Jiang XM. Conifer woods of the Pliocene age from Yunnan, China. *Journal of Integrative Plant Biology* 2005; **47(3)**: 264–70.


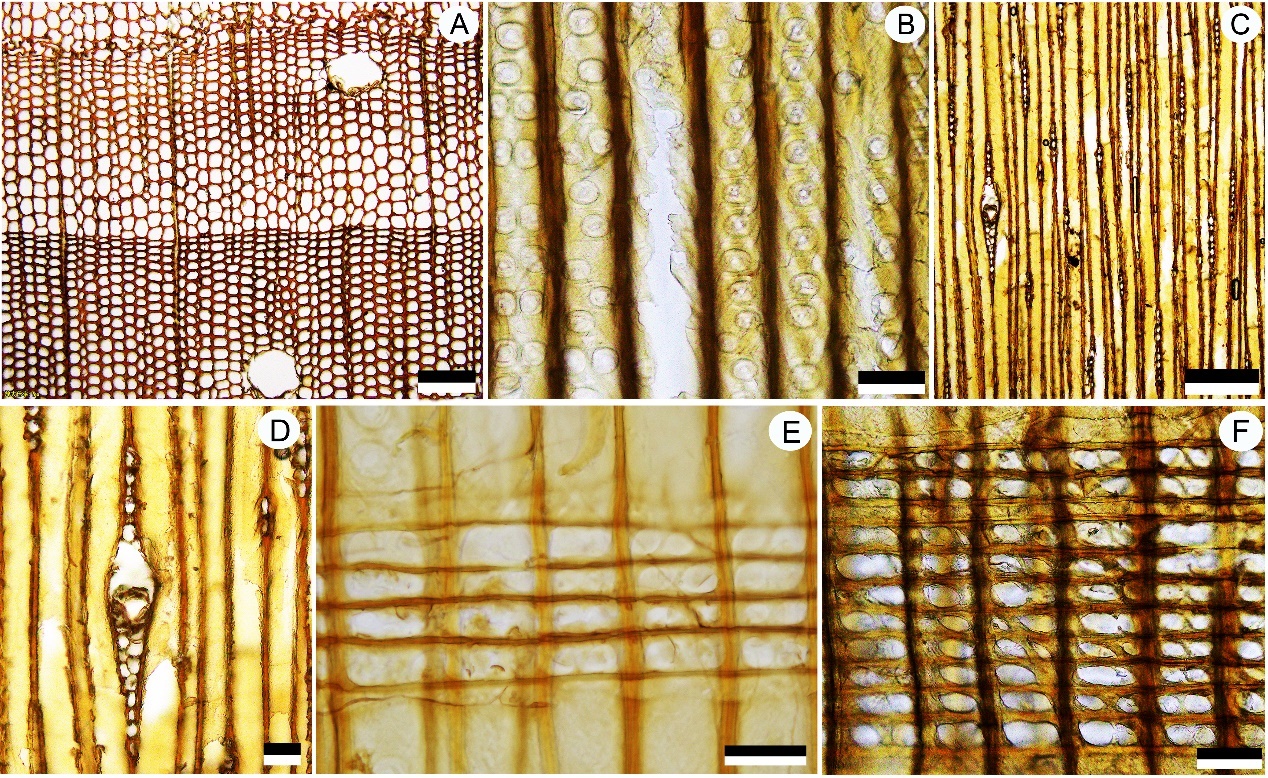


**Figure S1.** Wood structure of *Pinus armandii* from the Upper Pleistocene of Maoming. **A,** Transverse section showing distinct growth rings with gradual to somewhat abrupt transitions from earlywood to latewood; note axial resin canals occur in latewood. **B,** Tangential section; bordered pits on radial tracheid walls are uniseriate and biseriate (opposite); crassulae present. **C,** Tangential section; rays are mostly uniseriate and occasional fusiform. **D,** Showing fusiform rays in detail. **E,** Radial section; showing ray parenchyma with smooth horizontal walls and the end walls and ray tracheids occasionally in one tier at the margins of rays. **F,** Radial section; cross-field pits are mainly “window-like” (fenestriform), sometimes pinoid, 1–2 pits per cross field. Scale bars: 200 μm in **A**, **C**; 50 μm in **B**, **D–F**.


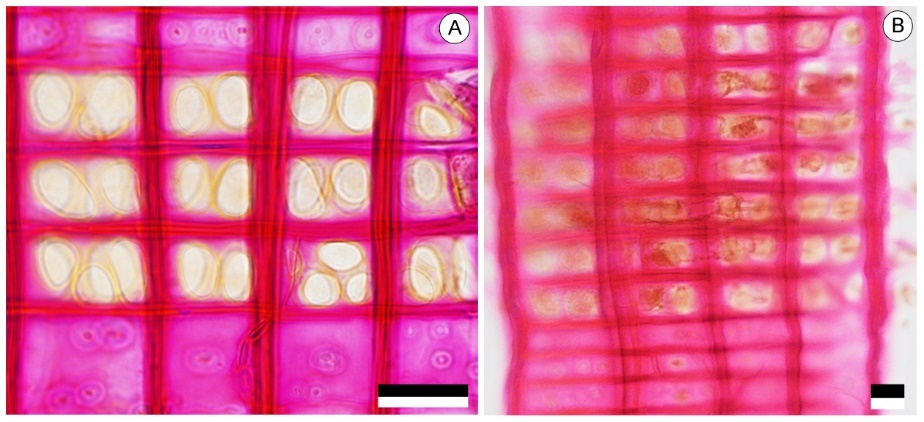


**Figure S2.** Features showing on cross-field in radial sections of extant woods of *Pinus koraiensis* and *Pinus armandii*. **A,** Cross-field pits arranged in two horizontal rows of *Pinus koraiensis*. **B,** In *Pinus armandii*, the end walls of its ray parenchyma cells are smooth, but few pits occasionally occur on their horizontal walls. Scale bars: 20 μm.


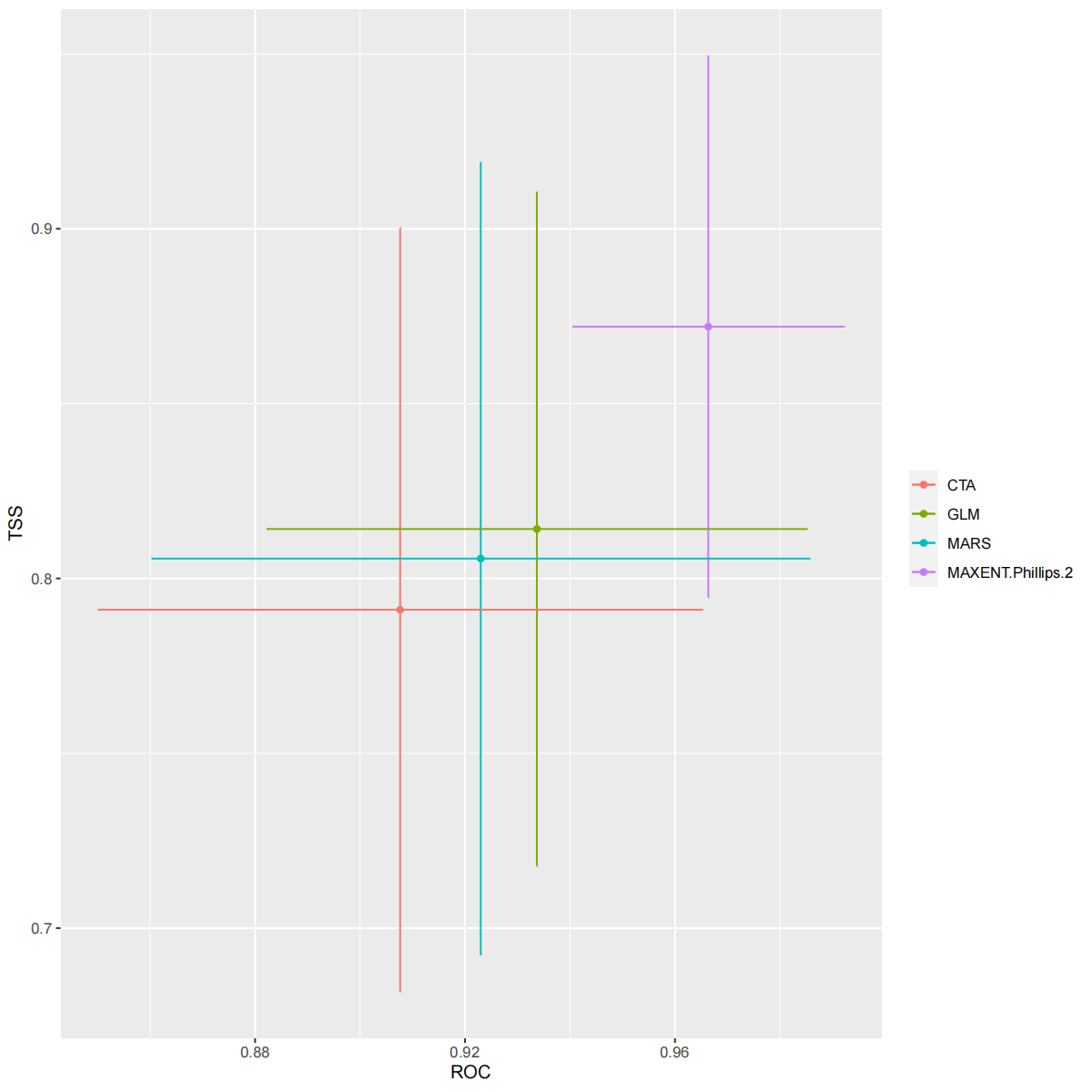


**Figure S3.** Mean model evaluation scores by algorithms according to ROC and TSS.


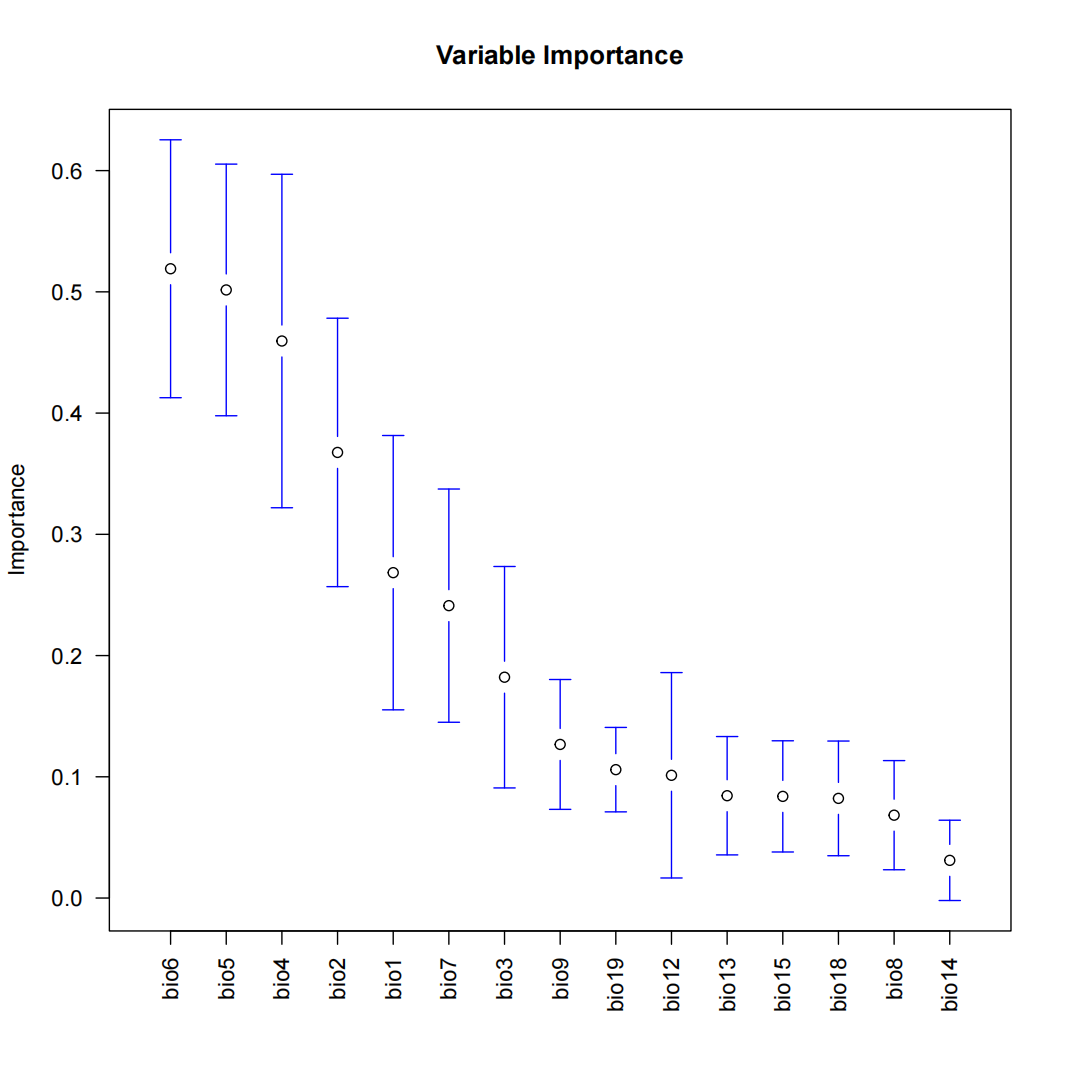
**Figure S4.** The 15 bioclimatic variables importance in the ensemble model.


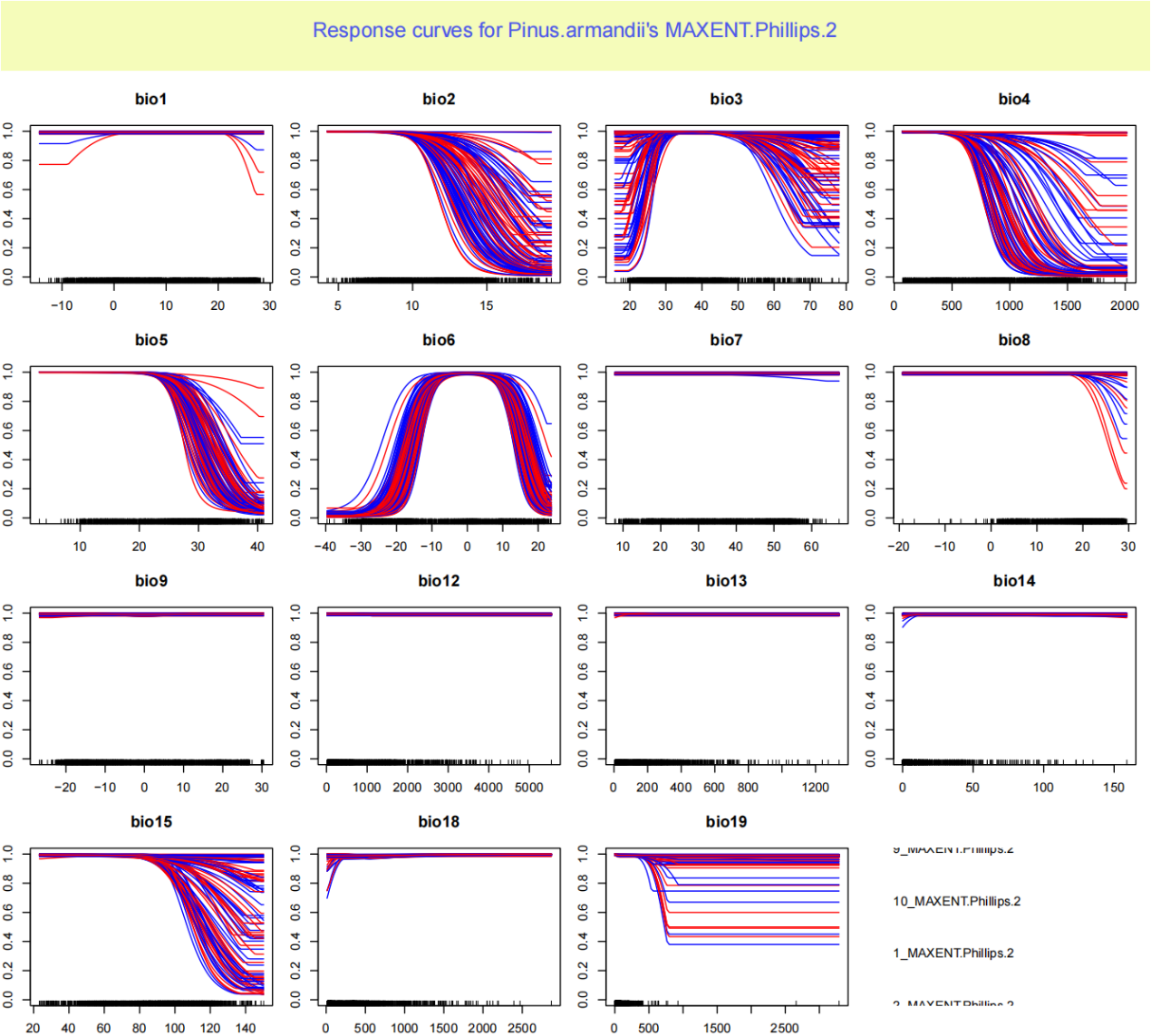


**Figure S5.** Response curves for the important environmental variables based on MaxEnt. Phillips.2.


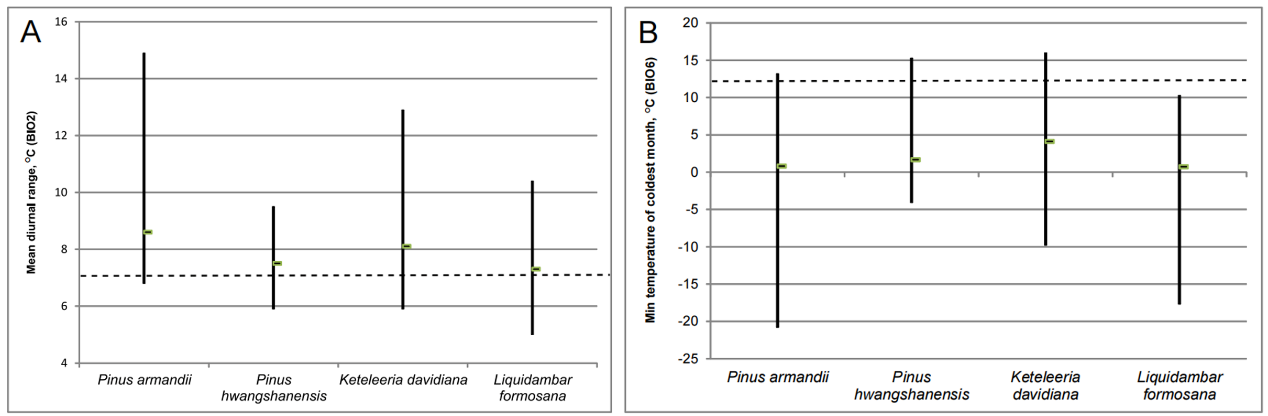


**Figure S6.** The ranges of two bioclimatic variables within the distribution areas of *Pinus armandii*, *Pinus* *hwangshanensis, Keteleeria davidiana* and *Liquidambar formosana*. **A,** Mean diurnal range, °C (BIO2). **B,** Minimum temperature of coldest month, °C (BIO6). Dashed line shows the modern values of bioclimatic variables for the fossil locality (21.86667°N; 110.66667°E) in the Maoming Basin, South China.

Table S1 Co­mparison of wood anatomical characters between *Pinus armandii* and extinct species of *Pinus.*

| Species | Location,  Age | Horizontal walls of ray tracheids | Tangential diameter of earlywood tracheid (μm) | Number of pit rows on radial walls of tracheids | Cross-field pitting | | Notes | References |
| --- | --- | --- | --- | --- | --- | --- | --- | --- |
|  |  |  |  |  | Numbers | Type |  |  |
| *Pinus armandii* Franch. | Maoming, China; Late Pleistocene | Smooth | 13–65 (mean 35.3) | 1–2 | 1–2 (2) | Window-like, pinoid |  | This paper |
| *Pinuxylon eutawense* Blackwell | Mississippi, USA; Late Cretaceous | Dentate | 30–76 (mean 55.4) | 1 | ? | Pinoid, occasional window-like |  | Blackwell, 1984 |
| *Pinus priamurensis* Blokhina et O.V. Bondarenko | Amur Region, Russia; late Middle Miocene–Late Miocene | Dentate | ? | 1 | 1–2 (1) | Window-like, pinoid |  | Blokhina and Bondarenko, 2016 |
| *Pinuxylon eschweilerense* van der Burgh | North Rhine-Westfalia, Germany; Pliocene | Dentate | 40 | 1 | 1–2 | Pinoid, window-like |  | Van der Burgh, 1973 |
| *Pinuxylon halepensoides* van der Burgh | North Rhine-Westfalia, Germany; Pliocene | Dentate to wavy | 25–30 | 1 | 1–2 | Piceoid, cupressoid |  | Van der Burgh, 1973 |
| *Pinuxylon paxii* Kräusel | North Rhine-Westfalia, Germany; Pliocene | Dentate | 25 | 1 | 1–2 | Window-like |  | Van der Burgh, 1973 |
| *Pinuxylon taedioides* Kräusel | Limburg, Netherlands; North Rhine-Westfalia, Germany; Pliocene | Distinctly dentate | 25–37 | 1–2 | 1–3 | Pinoid |  | Van der Burgh, 1964, 1973 |
| *Pinus henanensis* J.J. Yang | Henan, China; Cretaceous | Weakly dentate | Mainly 30–43 (up to 75) | 1–2 | 2–5 | Pinoid | Helical thickenings are present | Yang et al., 1990 |
| *Pinus uniseriata* H.B. Wang, A.A. Oskolski et Z.K. Zhou | Yunnan, China; Late Miocene | Smooth | 13–40 | 1–2 | 1–4 (6) | Taxodioid  and occasionally cupressoid |  | Wang et al., 2017 |
| *Pinus nanfengensis* H.B. Wang, A.A. Oskolski et Z.K. Zhou | Yunnan, China; Late Miocene | Weakly dentate to smooth | 15.0–37.9 | 1–2 | 1–6 | Pinoid and taxodioid |  | Wang et al., 2017 |
| *Pinuxylon arjuzanxianum* Huard | North Rhine-Westfalia, Germany; Pliocene | Weakly dentate | 25–30 | 1 | 1–4 | Pinoid |  | Van der Burgh, 1973 |
| *Pinuxylon tarnocziense* (Tuzson) Greguss | North Rhine-Westfalia, Germany; Pliocene | Weakly dentate | 30–40 | 1–2 | 2–3 | Pinoid |  | Van der Burgh, 1973 |
| *Pinuxylon ponderosoides* van der Burgh | North Rhine-Westfalia, Germany; Pliocene | Weakly dentate | 25 | 1 | 1–4 | Pinoid |  | Van der Burgh, 1973 |
| *Pinuxylon pinastroides* (Kraus) Stockmans et Willière | North Rhine-Westfalia, Germany; Pliocene | Weakly dentate | 25 | 1–2 | 1–4 | Cupressoid, taxodioid |  | Van der Burgh, 1973 |
| *Pinus* cf. *tabulaeformis* Carrière | Tegelen, Netherlands; Early Pleistocene | Weakly dentate (slender teeth) | 30 | 1 | 1–2 | Window-like |  | Van der Burgh, 1974 |
| *Pinus cembra* L. | Tegelen, Netherlands; Early Pleistocene | Weakly dentate | 43 | 1 | 1 | Window-like |  | Van der Burgh, 1974 |
| *Pinus driftwoodensis* Stockey | British Columbia, Canada; middle Eocene | Smooth, weakly dentate | 10–25 | 1 | 1–4 | Pinoid | The sample are branch | Stockey, 1983 |
| *Pinuxylon vateri* (Platen) Rössler | Oregon, USA; Miocene | Smooth to weakly dentate | ? | 1 | 2–4 | Taxodioid |  | Rössler, 1937 |
| *Pinus hatamuraenase* Jeong et Kim | Akita Prefecture, Japan; Middle Miocene | Smooth to weakly dentate | 48–52 | 1–2 | 1–2 | Pinoid |  | Jeong et al., 2012 |
| *Pinuxylon parryoides* (Kräusel) van der  Burgh | Limburg, Netherlands; North Rhine-Westphalia, Germany; Pliocene | Smooth, weakly dentate | 35–45 | 1–2 | (1) 2–4 (5) | Pinoid |  | Van der Burgh, 1964, 1973 |
| *Pinus* cf. *armandii* Franchet | Yunnan, China; Pliocene | Smooth to weakly dentate | Up to 45 | 1–2 | 1–4 | Window-like, occasional pinoid |  | Yi et al., 2002 |
| *Pinuxylon chemrylensis* Blokhina | Kamchatka, Russia; Paleocene-early Eocene | Smooth | ? | 1 | 2–3 (4) | Piceoid, cupressoid, taxodioid |  | Blokhina, 1995 |
| *Pinus similkameenensis* Miller | British Columbia, Canada; middle Eocene | Smooth | 10–30 | 1 | 1–4 | Pinoid |  | Miller, 1973 |
| *Pinuxylon woolardii* Tidwell, Parker et Folkman | Eastern Oregon, USA; Miocene | Smooth | ? | 1–2 | 2–5 | Taxodioid, cupressoid (pinoid) |  | Tidwell et al., 1986 |
| *Pinuxylon zobelianum*  (Goeppert) Kräusel | Limburg, Netherlands; Miocene | Smooth | 18–30 | 1–2 | 1–2 (3) | Window-like |  | Van der Burgh, 1964 |
| *Pinus albicauloides* S.K.  Choi et K. Kim | Yamagata Prefecture, Japan; Miocene | Smooth | 25–70 | 1–2 | 1–2 | Window-like |  | Choi et al., 2010 |
| *Pinuxylon cembraeforme* Rössler | Steiermark, Austria; Pliocene | Smooth | ? | 1 | 1 | Window-like |  | Rössler, 1937 |
| *Pinus mundayi* Falcon-Lang, Mages, Collinson | Nova Scotia, Canada; Early Cretaceous | No ray tracheids | 10–25 | 1–2 | 1–4 | Window-like or pinoid | Charred long shoots | Falcon-Lang et al., 2016 |
| *Pinuxylon microporosum* Ogura | Hokkaido, Japan; Saghalien, Russia; Early Cretaceous | No ray tracheids | ? | 1–2 | 1–3 | Pinoid, piceoid |  | Ogura, 1944; Nishida and Nishida, 1995 |
| *Pinuxylon* sp. | Inner Mongolia, China; Miocene | No ray tracheids | ? | 1 | 1 | Window-like |  | Tao et al., 1994 |

Table S2 Average, minimum and maximum values of bioclimatic variables (BIO1–BIO19) for *Pinus armandii* and other species represented by fossil woods in the Late Pleistocene deposits of the Maoming Basin. Temperature variables are degrees Celcius × 10.

| Bioclimatic Variables | Value | *Keteleeria davidiana* | *Liquidambar formosana* | *Pinus armandii* | *Pinus hwangshanensis* |
| --- | --- | --- | --- | --- | --- |
| BIO1 | min | 62 | 108 | -18 | 103 |
|  | average | 172 | 196 | 119 | 140 |
|  | max | 242 | 258 | 270 | 222 |
| BIO2 | min | 59 | 50 | 68 | 65 |
|  | average | 81 | 73 | 86 | 75 |
|  | max | 129 | 104 | 149 | 80 |
| BIO3 | min | 23 | 22 | 23 | 22 |
|  | average | 32 | 35 | 44 | 25 |
|  | max | 52 | 53 | 53 | 33 |
| BIO4 | min | 2622 | 2637 | 2475 | 5723 |
|  | average | 6165 | 5082 | 3995 | 7741 |
|  | max | 9004 | 9152 | 9605 | 8732 |
| BIO5 | min | 163 | 178 | 122 | 243 |
|  | average | 295 | 298 | 206 | 283 |
|  | max | 336 | 348 | 380 | 337 |
| BIO6 | min | -98 | -39 | -208 | -53 |
|  | average | 41 | 83 | 9 | -11 |
|  | max | 160 | 175 | 132 | 95 |
| BIO7 | min | 141 | 141 | 145 | 233 |
|  | average | 254 | 215 | 198 | 294 |
|  | max | 358 | 339 | 385 | 325 |
| BIO8 | min | 120 | 139 | 56 | 181 |
|  | average | 233 | 244 | 161 | 226 |
|  | max | 281 | 294 | 285 | 286 |
| BIO9 | min | -5 | 26 | -101 | 16 |
|  | average | 96 | 146 | 75 | 53 |
|  | max | 212 | 273 | 237 | 158 |
| BIO10 | min | 120 | 141 | 62 | 202 |
|  | average | 247 | 255 | 165 | 236 |
|  | max | 285 | 294 | 307 | 286 |
| BIO11 | min | -5 | 14 | -101 | -3 |
|  | average | 89 | 127 | 64 | 37 |
|  | max | 202 | 220 | 215 | 141 |
| BIO12 | min | 627 | 646 | 249 | 1313 |
|  | average | 1713 | 2049 | 2332 | 1663 |
|  | max | 3971 | 3790 | 4192 | 2014 |
| BIO13 | min | 134 | 87 | 73 | 200 |
|  | average | 300 | 358 | 391 | 269 |
|  | max | 896 | 922 | 918 | 329 |
| BIO14 | min | 2 | 4 | 0 | 32 |
|  | average | 45 | 45 | 51 | 42 |
|  | max | 170 | 214 | 122 | 51 |
| BIO15 | min | 33 | 12 | 36 | 49 |
|  | average | 65 | 64 | 67 | 54 |
|  | max | 103 | 106 | 124 | 65 |
| BIO16 | min | 347 | 257 | 182 | 555 |
|  | average | 800 | 971 | 1059 | 718 |
|  | max | 2336 | 2294 | 2463 | 841 |
| BIO17 | min | 10 | 17 | 2 | 114 |
|  | average | 150 | 159 | 175 | 155 |
|  | max | 543 | 773 | 383 | 191 |
| BIO18 | min | 322 | 203 | 180 | 508 |
|  | average | 728 | 899 | 1005 | 694 |
|  | max | 2336 | 2294 | 2463 | 841 |
| BIO19 | min | 10 | 17 | 2 | 114 |
|  | average | 167 | 196 | 232 | 171 |
|  | max | 667 | 921 | 749 | 219 |

Notes: BIO1: Annual mean temperature (BIO1), °C; BIO2: Mean diurnal range, °C; BIO3: Isothermality (BIO3), %; BIO4: Temperature seasonality (standard deviation), °C; BIO5: Max. temperature of warmest month, °C; BIO6: Min. temperature of coldest month, °C; BIO7: Temperature annual range, °C; BIO8: Mean temperature of wettest quarter, °C; BIO9: Mean temperature of driest quarter, °C; BIO10: Mean temperature of warmest quarter, °C; BIO11: Mean temperature of coldest quarter, °C; BIO12: Annual precipitation, mm; BIO13: Precipitation of wettest month, mm; BIO14: Precipitation of driest month, mm; BIO15: Precipitation seasonality (coefficient of variation), %; BIO16: Precipitation of wettest quarter, mm; BIO17: Precipitation of driest quarter, mm; BIO18: Precipitation of warmest quarter, mm; BIO19: Precipitation of coldest quarter, mm.

Table S3 The ranges of some bioclimatic variables for the Late Pleistocene (33–30 ka BP) of Maoming Basin reconstructed by their variation within the distribution areas of four co-occurred species (*Pinus armandii*, *Pinus* *hwangshanensis,* *Keteleeria davidiana* and *Liquidambar formosana*) compared with the results of past climate modelling by Oscillayers (ages 30 ka BP and 20 ka BP, 22.5°N, 112.5°E ) and the WorldClim data for the climate at the fossil locality (21.86667°N; 110.66667°E) during the Last Glacial Maximum (21 ka BP), Mid-Holocene (6 ka BP), and the present time.

| Climate  Variables | 33–30 ka | 30 ka | 20 ka | 21 ka | 6 ka | Modern |
| --- | --- | --- | --- | --- | --- | --- |
|  | Coexistence Approach  (4 species) | Oscillayers | Oscillayers |  |  |  |
| Annual mean temperature, ℃ (BIO1) | 10.8−22.2 | 19.24 | 19.97 | 19.98 | 23.18 | 23.20 |
| Mean diurnal range, ℃ (BIO2) | 6.8−7.9 | 8.73 | 8.44 | 8.43 | 7.33 | 7.10 |
| Isotermality (BIO3) | 23−33 | 36.67 | 38.22 | 38.11 | 30.89 | 34.00 |
| Temperature seasonality (BIO4) | 5723−8732 | 5356.89 | 4937.78 | 4946.67 | 5424.22 | 4827.00 |
| Maximum temperature of warmest month, ℃ (BIO5) | 24.3−33.6 | 29.93 | 29.76 | 29.74 | 34.04 | 32.40 |
| Minimum temperature of coldest month, ℃ (BIO6) | -3.9−9.5 | 6.43 | 7.93 | 7.90 | 10.68 | 11.80 |
| Temperature annual range, ℃ (BIO7) | 23.3−32.5 | 23.47 | 21.82 | 21.82 | 23.36 | 20.60 |
| Mean temperature of wettest quarter, ℃ (BIO8) | 18.1−28.1 | 23.99 | 25.67 | 25.70 | 29.62 | 28.60 |
| Mean temperature of driest quarter, ℃ (BIO9) | 2.6−15.8 | 15.31 | 13.96 | 13.99 | 15.73 | 18.00 |
| Mean temperature of warmest quarter, ℃ (BIO10) | 20.2−28.5 | 25.42 | 25.67 | 25.70 | 29.62 | 28.60 |
| Mean temperature of coldest quarter, ℃ (BIO11) | 1.4−14.1 | 11.71 | 12.98 | 13.00 | 15.73 | 16.40 |
| Annual precipitation, mm (BIO12) | 1313−2014 | 1513.56 | 1404.56 | 1404.33 | 1734.33 | 1629.00 |
| Precipitation of wettest month, mm (BIO13) | 200−329 | 262.56 | 259.78 | 259.56 | 287.33 | 306.00 |
| Precipitation of driest month, mm (BIO14) | 32−51 | 20.22 | 15.33 | 15.22 | 23.33 | 25.00 |
| Precipitation seasonality (BIO15) | 49−65 | 73.33 | 79.33 | 79.22 | 65.22 | 71.00 |
| Precipitation of wettest quarter, mm (BIO16) | 555−841 | 729.44 | 714.44 | 713.89 | 744.56 | 795.00 |
| Precipitation of driest quarter, mm (BIO17) | 114−191 | 81.22 | 62.33 | 62.00 | 93.44 | 98.00 |
| Precipitation of warmest quarter, mm (BIO18) | 508−841 | 692.67 | 714.44 | 714.00 | 744.56 | 795.00 |
| Precipitation of coldest quarter, mm (BIO19) | 114−219 | 122.44 | 75.44 | 75.67 | 93.56 | 101.00 |
